# Design and Application of Atomically Dispersed Transition Metal–Carbon Cathodes for Triggering Cascade Oxygen Reduction in Wastewater Treatment

**DOI:** 10.3390/molecules30153258

**Published:** 2025-08-04

**Authors:** Shengnan Huang, Guangshuo Lyu, Chuhui Zhang, Chunye Lin, Hefa Cheng

**Affiliations:** 1 State Key Laboratory of Water Environment Simulation, School of Environment, Beijing Normal University, Beijing 100875, China; huangshnan@163.com (S.H.); c.lin@bnu.edu.cn (C.L.); 2 MOE Laboratory for Earth Surface Process, College of Urban and Environmental Sciences, Peking University, Beijing 100871, China; gslyu@pku.edu.cn; 3 School of Water Resources and Environment, China University of Geosciences (Beijing), Beijing 100083, China; czhang24@cugb.edu.cn

**Keywords:** single-atom electrocatalyst, heterogeneous electro-Fenton process, cascade oxygen reduction reaction, wastewater treatment, atomically engineered cathodes, electrochemical oxidation, reactive oxygen species, catalyst stability

## Abstract

The precise synthesis of non-precious metal single-atom electrocatalysts is crucial for enhancing the yield of highly active reactive oxygen species (ROSs). Conventional oxidation methods, such as Fenton or NaClO processes, suffer from poor efficiency, high energy demand, and secondary pollution. In contrast, heterogeneous electro-Fenton systems based on cascade oxygen reduction reactions (ORRs), which require low operational voltage and cause pollutant degradation through both direct electron transfer and ROS generation, have emerged as a promising alternative. Recent studies showed that carbon cathodes decorated with atomically dispersed transition metals can effectively integrate the excellent conductivity of carbon supports with the tunable surface chemistry of metal centers. However, the electronic structure of active sites intrinsically hinders the simultaneous achievement of high activity and selectivity in cascade ORRs. This review summarizes the advances, specifically from 2020 to 2025, in understanding the mechanism of cascade ORRs and the synthesis of transition metal-based single-atom catalysts in cathode electrocatalysis for efficient wastewater treatment, and discusses the key factors affecting treatment performance. While employing atomically engineered cathodes is a promising approach for energy-efficient wastewater treatment, future efforts should overcome the barriers in active site control and long-term stability of the catalysts to fully exploit their potential in addressing water pollution challenges.

## 1. Introduction

With rapid industrialization and intensified agricultural activity, the accumulation of toxic organic pollutants in aquatic ecosystems has become a severe global environmental challenge [1]. Antibiotics, pesticides, and halogenated compounds released into the environment exhibit inherent toxicity, strong bioaccumulation potential, and environmental persistence, thereby posing serious risk to both ecosystem and human health [1,2,3,4,5]. Moreover, many organic pollutants are recalcitrant during conventional water treatment processes, resulting in their accumulation in the receiving water bodies and exacerbating ecological risks [6,7]. These challenges underscore the urgent need for the development of innovative, efficient, and environmentally friendly technologies for the treatment of these pollutants. In recent years, electrochemical advanced oxidation processes (EAOPs) have emerged as a promising solution. Compared to conventional methods, they offer several advantages, including higher treatment efficiency, more compact system design, and enhanced adaptability to diverse wastewater types. Furthermore, EAOPs often have lower chemical consumption, energy demand, and overall environmental impact [8,9,10]. Several electrochemical technologies have already been successfully implemented in the treatment of industrial and domestic wastewater, achieving satisfactory performance under field conditions [11,12]. Meanwhile, a range of new electrochemical treatment approaches, based on novel reaction systems or electrode materials, have been proposed and validated at the pilot scale [13,14]. Therefore, electrocatalytic wastewater treatment is increasingly recognized as one of the most promising and suitable technologies to address water pollution.

**Figure 1:**
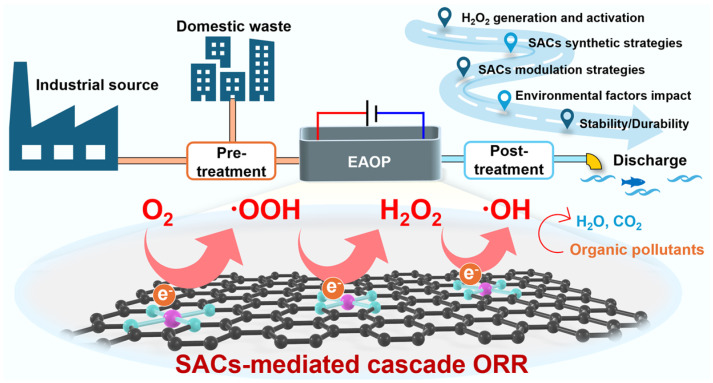
Schematic illustration of SAC-mediated cascade ORR.

The cascade oxygen reduction reaction (ORR), an enhanced solution for the conventional heterogeneous electro-Fenton system, is a newly proposed cathodic EAOP that has attracted increasing attention due to its distinct advantages (Figure 1) [15,16,17,18,19,20,21]. While the heterogeneous electro-Fenton system effectively addresses the challenge of metal-ion recovery in the homogeneous Fenton process and enables the reduction of O_2_ to H_2_O_2_ and subsequently to ^•^OH via interfacial interactions, it still has some limitations. Specifically, conventional catalysts, such as metal oxides and zero-valent metal nanoparticles, have their active metal centers confined to the catalyst surface. The yield of ROSs is thus restricted by the redox cycling rate of metal ions, and excessive metal leaching during the reduction process may cause secondary pollution. In contrast to the conventional anodic oxidation process that requires high overpotential (above 2.5 V), the cascade ORR proceeds at substantially lower potential (0.5–1.2 V), thereby reducing energy consumption [22,23]. For instance, the Cu,N co-doped carbon system designed by Zhou et al. exhibited an energy consumption of only 1.1 kWh/g TOC, which is comparable to that of competitive UV/H_2_O_2_ and H_2_O_2_/O_3_ systems [24,25]. A key feature of this process is the utilization of O_2_ from ambient air as the sole oxidant precursor. O_2_ can be activated to generate reactive species, such as hydrogen peroxide (H_2_O_2_) and superoxide radicals (^•^O_2_^−^), via the ORR, eliminating the need for externally added chemical oxidants and the production of ferric sludge [26,27,28]. Atomically dispersed catalysts downsize the metal particles to the atomic scale, allowing for the modulation of the coordination environment between metal atoms and a support to lower the reaction energy barrier, while simultaneously improving the utilization efficiency of metal atoms. Moreover, when carbon-based materials are used as the support, the electrical conductivity of the catalyst is significantly enhanced, further reducing the intrinsic impedance of the cathode material. Notably, it has been shown that employing N-doped carbon felts, ZIF-67-NC/CB and Ni_0.41_Fe_0.39_@NC, as the cathode materials can effectively suppress the formation of toxic products from pollutant degradation and resistance genes during electrochemical treatment, thereby lowering the environmental risk [29,30,31].

To enhance the activity and selectivity of carbon materials, atomic modification with transition metals has emerged as an effective strategy for optimizing the electronic structure and increasing the number of active sites. Carbon materials serve as ideal supports for anchoring these active sites due to their high electrical conductivity, large surface areas, and tunable surface chemistry [32,33]. When transition metals, such as Fe, Ni, and Co, are introduced, their d-orbital electron can hybridize with the carbon substrate, effectively lowering the reaction energy barrier for the ORR [34,35,36]. Additionally, the effect induced by the defects and heteroatom doping (e.g., N, S, and B) on the carbon substrate can further improve the dispersion and stability of surface active sites [37,38,39,40]. While metal-free catalysts have occasionally been explored, transition metal-based catalysts show greater potential due to their high activity and pollutant degradation efficiency [41]. For instance, Fe_3_C and FeN_x_ sites can efficiently activate O_2_ to generate H_2_O_2_ at a potential of 0.6 V (vs. NHE), subsequently triggering Fenton-like reactions to produce ^•^OH for the degradation of various chlorophenols [42].

Transition metal-based single-atom electrocatalysts (SACs) have been extensively explored and commercially applied in fuel cells and metal–air batteries due to their outstanding ORR performance, which inspires their application in the field of environmental remediation [43,44,45,46,47]. However, their environmental application remains at an early stage due to the unclear electron transfer processes in the cascade ORR and challenges in precisely controlling the structure of active sites. For instance, a ZIF nanocage, which can trap various transition metal ions through amine ligands, is an ideal precursor for the synthesis of SACs [48,49,50]. Distinct catalytic properties can be obtained by adjusting the metal centers, and pyrolysis temperature and time. It has been shown that Co/Fe-NC derived from ZIF-8 could effectively regulate electron transfer, thereby enhancing ^•^OH selectivity [16], while Fe-Cu/NC could simultaneously facilitate both dechlorination and oxidation processes [31]. Meanwhile, Zhang et al. reported that Ni/Fe-NC brought simultaneous reduction and oxidation reactions, underscoring the complexity of electron transfer and active site interaction [51]. Additionally, different synthesis approaches further complicate the structure–performance relationship of the catalyst. Therefore, further efforts are needed to elucidate reaction mechanisms at the atomic level and to design robust SACs with improved selectivity, stability, and real-world applicability.

This review provides a comprehensive overview of recent advances in transition metal SACs for electrocatalytic water treatment, focusing on synthesis and modulation strategies, reaction mechanisms, the influence of operational conditions on treatment performance, and approaches to mitigate catalyst deactivation and extend service life. It is intended for researchers engaged in the following: (i) understanding the underlying mechanisms of the cascade ORR; (ii) development of cathodic materials in electrocatalytic systems; (iii) precise design and regulation of SACs for the ORR; (iv) investigation of key environmental factors affecting electrocatalytic performance; and (v) investigation of cathodic catalyst deactivation. The content is intended to guide future research directions and promote the rational design of efficient cathodic materials for environmental applications.

## 2. Mechanism and Performance Evaluation of Cascade ORR

### 2.1. Fundamental Principles of Cascade ORR

**Figure 2:**
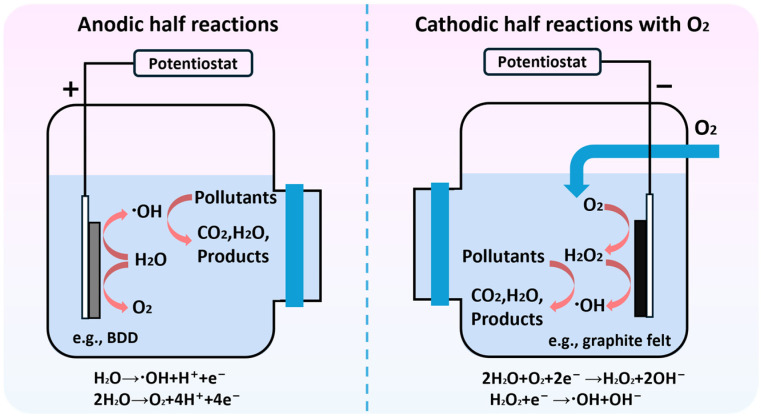
Schematic illustration of the degradation mechanisms of organic pollutants at the anode and cathode.

A major scientific challenge in the design and application of transition metal SACs for electrocatalytic water treatment is to achieve directional and efficient generation of ROSs via SACs [52]. Figure 2 illustrates a typical electrocatalytic cascade ORR system and its main reaction pathways. For the conventional electro-Fenton process, its rate-determining step is commonly the generation and activation of H_2_O_2_ [53]. Specifically, H_2_O_2_ is first produced via a 2e^−^ ORR (Equation (1)), and then activated through the Haber–Weiss cycle and related pathways to generate ^•^OH (Equations (2)–(7)) [54]. In contrast, the cascade ORR enables two-step electron transfer to simultaneously generate and activate ROSs (Equations (1) and (8)), significantly improving the utilization efficiency of electrons.

2e^−^ ORR:

(1) $$O_{2}+2H^{+}+2e^{-}\to H_{2}O_{2}$$

Habor–Weiss cycle:

(2) $$M_{surface}^{n+}+H_{2}O_{2}\to M_{surface}^{\left(n+1\right)+}\cdot OH+{OH}^{-}$$

(3) $$M_{surface}^{\left(n+1\right)+}+H_{2}O_{2}\to M_{surface}^{n+}\cdot OOH+H^{+}$$

(4) $$M_{surface}^{n+}+H_{2}O_{2}\to M_{surface}^{\left(n+2\right)+}+2{OH}^{-}$$

(5) $$M_{surface}^{\left(n+2\right)+}+H_{2}O_{2}\to M_{surface}^{n+}+O_{2}+2H^{+}$$

(6) $$2\cdot OH+M_{surface}^{n+}\to M_{surface}^{\left(n+2\right)+}+2{OH}^{-}$$

(7) $$M_{surface}^{\left(n+1\right)+}+e^{-}\to M_{surface}^{n+}$$

H_2_O_2_ reduction:

(8) $$H_{2}O_{2}+e^{-}\to \cdot OH+{OH}^{-}$$

Table 1 summarizes the recent studies on degradation of emerging contaminants by the electrocatalytic cascade ORR. Organic compounds with electron-withdrawing groups typically exhibit high ionization potentials (IPs) and are prone to be oxidized by radical species, whereas those with electron-donating groups have lower IPs and are more readily oxidized by non-radical species [55]. As shown in Table 1, certain active site configurations can effectively facilitate the generation of highly oxidative ^•^OH during the oxygen reduction process mediated by atomically dispersed catalysts, while others can mediate the production of highly selective ^1^O_2_ or ^•^O_2_^−^, both of which can contribute to the mineralization of organic contaminants [56]. However, H_2_O_2_ will undergo disproportionation to form O_2_ and H_2_O when Ce serves as the metal center, thereby reducing the selectivity toward ^•^OH generation [57]. These findings highlight the critical role of metal centers in determining ROS generation pathways and catalytic performance.

### 2.2. Generation of H_2_O_2_

**Figure 3:**
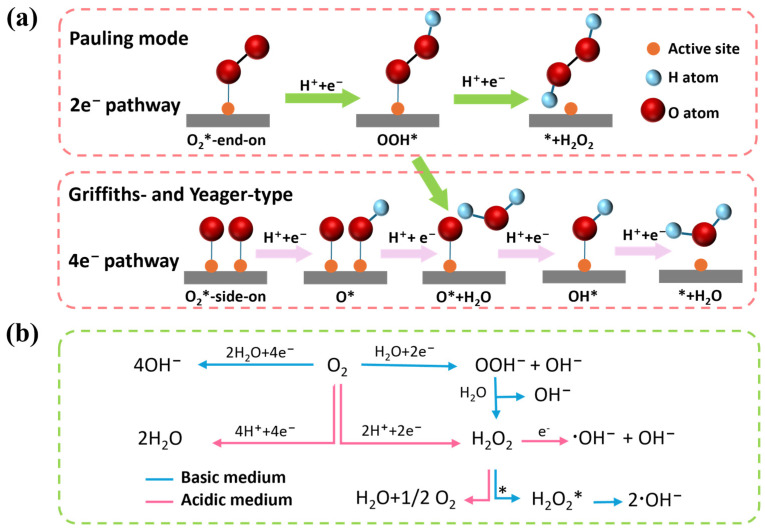
Schematic illustrations for (**a**) the adsorption and activation of oxygen molecules, and (**b**) the ORR pathway mediated by SACs (* represents the active sites).

The 2e^−^ ORR process is of particular interest in environmental applications because it enables the in situ generation of H_2_O_2_. However, the electrocatalytic 2e^−^ ORR process, which is often accompanied by competing 4e^−^ reduction pathways that lead to water formation, typically requires high overpotential due to sluggish reaction kinetics. Therefore, the development of SACs with high efficiency and selectivity toward the 2e^−^ ORR pathway is of great significance for advancing water treatment technologies [77,78]. As shown in Figure 3a, the adsorption mode of O_2_ determines the activation pathway. Pauling-type (end-on) adsorption of O_2_ at metal sites is highly favorable for the selective 2e^−^ ORR, as this binding mode preserves the O-O bond and promotes the formation of H_2_O_2_ [79]. In contrast, Griffiths- and Yeager-type (side-on) adsorption configurations tend to facilitate O-O bond cleavage, favoring the 4e^−^ ORR pathway that leads to water formation [80,81]. Due to the absence of contiguous active sites on a single-atom surface, bridge-like adsorption configurations are not feasible, which prevents the occurrence of O-O bond cleavage [82].

The formation of intermediate adsorbed oxygen species on the active sites plays a critical role in determining the products of the ORR. Typically, in the associative oxygen reduction pathway, O_2_ is initially adsorbed onto the single-atom active sites through the Pauling mode, with the O-O bond aligning with the Z-axis of the metal center. This adsorption configuration facilitates the stepwise electrochemical protonation of O_2_ to form the OOH* intermediate. Subsequently, the OOH* intermediate can either undergo 2e^−^ reduction to yield H_2_O_2_, or dissociate into O* and H_2_O, initiating the 4e^−^ pathway toward complete oxygen reduction [79]. The adsorption free energy of the OOH* intermediate is widely recognized as a reliable descriptor to evaluate ORR activity for both 2e^−^ and 4e^−^ pathways [83]. To minimize the occurrence of the 4e^−^ process, the adsorption of OOH* should be strengthened, whereas the adsorption of O*, a dissociation product of OOH*, should be weakened [82]. However, modulating the binding strength of O* often changes the OOH* adsorption energy, which may compromise the selectivity of H_2_O_2_. Numerous studies have demonstrated that atomic dispersion of metal species on 2D materials provides an effective strategy to balance this process, enabling the development of cost-effective, efficient, stable, and highly selective electrocatalysts for the direct synthesis of H_2_O_2_ [84,85].

Solution pH significantly influences the adsorption of reaction intermediates on the active sites (Figure 3b). In acidic media, the strong protonation of intermediates can promote the formation of OOH* [65]. However, excessive acidity tends to drive the reaction toward the 4e^−^ pathway as shown in Figure 3a, leading to the generation of H_2_O. In alkaline media, the low proton concentration increases the energy barrier for the dissociation of OOH* into O* and H_2_O, while enhancing the tendency for desorption to form H_2_O_2_, thereby favoring the 2e^−^ pathway. Meanwhile, the generated H_2_O_2_ is more prone to dismutate into ^•^OH in alkaline media, whereas it tends to decompose into H_2_O and O_2_ in acidic media.

### 2.3. Performance Evaluation

The electron transfer number (n) is commonly used to evaluate the selectivity of the catalyst toward the 2e^−^ or 4e^−^ pathway. Wan et al. summarized the methods employed for measuring and calculating the n value based on the rotating ring-disk electrode (RRDE) method [86]. Notably, in RRDE experiments, the ring potential is typically set at 1.23 V (vs. RHE) to ensure that generated H_2_O_2_ is completely oxidized to O_2_. It is essential to calibrate the collection efficiency (N_c_), which represents the ratio between the amount of H_2_O_2_ oxidized on the Pt ring and H_2_O_2_ generated on the glass carbon electron disk [87]. This value can be experimentally determined using the single-electron redox couple of Fe(CN)_6_^3−^/Fe(CN)_6_^4−^ [27,88]. For platinum group metal-free (PGM-free) materials, the main ORR performance parameters include the electrochemical surface area (ECSA), the electrode-area-normalized current density (j_es_) at 0.85 V (vs. RHE), and the half-wave potential (E_1/2_), which represents the potential at which the current reaches half of its diffusion-limited value and is a key indicator of catalytic kinetics. The Tafel slope is also a crucial parameter for assessing the reaction mechanism [86,89]. Additionally, the population of the catalytically active sites and the ORR rate are also crucial parameters to be considered [90,91]. These metrics collectively provide a comprehensive evaluation of both catalytic activity and pathway selectivity and ultimately guide the rational design of high-performance electrocatalysts for practical applications.

A unified metric for describing H_2_O_2_ selectivity is crucial for advancing research in this field. Current indicators include molar fraction selectivity and Faradaic selectivity. However, most existing studies either fail to report these parameters or lack consistency in their application. Xia et al. advocated for the use of Faradaic efficiency (FE) as the standard metric for reporting H_2_O_2_ selectivity in electrocatalysis, rather than molar fraction selectivity, due to the inconsistency in H_2_O_2_ selectivity [92]. In RRDE measurement, molar fraction selectivity ($X_{H_{2}O_{2}}$) can overestimate the performance by up to 17.2% compared to FE. The relationship is defined with Equation (9). As FE directly measures current efficiency, it accounts for competing reactions (e.g., hydrogen evolution and catalyst oxidation), whereas the molar fraction assumes only H_2_O_2_ and H_2_O to be the products. FE is also widely adopted in other electrosynthesis fields (e.g., CO_2_ reduction), ensuring cross-disciplinary clarity. Therefore, reporting the selectivity metric (FE or molar fraction) explicitly is crucial to avoid misinterpretation, especially when comparing the RRDE data with bulk electrolysis results.

(9) $$X_{H_{2}O_{2}}=\frac{2\cdot FE}{1+FE}$$

## 3. Design and Modulation of Active Sites in Cathode Materials

### 3.1. Synthesis Strategies for Atomically Dispersed Catalysts

**Figure 4:**
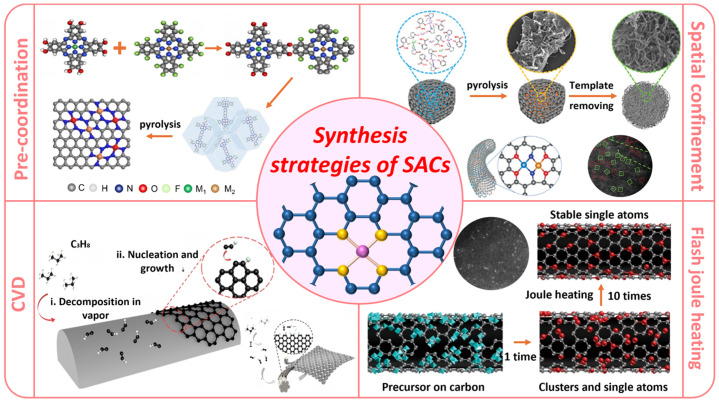
Summary of the major strategies for the synthesis of atomically dispersed catalysts (adapted from [100,101,102,103]).

Extensive efforts have been made toward downsizing metallic nanomaterials to single atoms, aiming to expose more active sites, thereby boosting electrocatalytic performance. However, the synthesis of SACs remains a formidable challenge, largely due to the intrinsic tendency of isolated metal atoms to aggregate into nanoparticles, which is driven by their high surface free energy [93]. Establishing strong metal–support interaction (SMSI) has played a pivotal role in the design of SACs [94]. Strong bonding between metal atoms and a support not only inhibits atom migration but also enables modulation of the local electronic structure via charge transfer between the metal center and coordinating atoms, both of which are critical for enhancing catalyst stability [95]. Therefore, rational synthesis strategies are essential to boost catalytic performance and ensure durability under harsh reaction conditions. For the carbon support, both synthetic carbon materials, such as carbon nanotubes, graphene, and carbon felt, and biochar derived from natural sources can be used. The most notable and forward-looking approaches currently employed to obtain atomically dispersed catalysts include MOF-based ligand-assisted pre-anchoring, spatial confinement, chemical vapor deposition (CVD), and carbon-assisted flash Joule heating (Figure 4) [93,96,97,98,99].

#### 3.1.1. Pre-Coordination Strategy

The core principle of this strategy is to suppress the metal atom aggregation during high-temperature treatment by employing strong interactions, such as coordination, electrostatic adsorption, and π-π stacking, between the metal species and functional groups embedded within the substrate. Potassium thiocyanate [104], saloph [105], and 5-(2-pyridyl)-1H-tetrazole [106] can form robust coordination complexes with metal atoms, effectively minimizing atomic migration and suppressing metal atom aggregation during high-temperature synthesis. In addition, functional groups can be tailored on the substrate or chelating ligands to construct strong metal–heteroatom coordination structures [107]. Chelating structures with dual-ligand configurations also provide a promising approach for the controlled fabrication of dual-atom catalysts.

Tang et al. [96] employed bidentate ligands (phenanthroline-functionalized covalent organic polymers, Phen-COP) along with dual bidentate ligands (Phen-COP and 2,2′-bipyridine) to pre-anchor Co atoms, enabling precise modulation of the first-shell coordination environment around single Co atoms. Subsequent high-temperature pyrolysis successfully yielded asymmetric low-coordination Co-N_3_ and high-coordination Co-N_4_ active sites anchored on carbon nanotube support. Metal phthalocyanines also provide a structurally well-defined platform for the fabrication of atomically dispersed catalysts. Functionalization of the peripheral aromatic rings with electron-withdrawing substituents enables selective coupling with nucleophilic ligands, thereby enabling controlled molecular assembly and precise integration of dual-metal sites. Rao et al. [100] proposed this versatile approach for the synthesis of dual-atom catalysts (DACs), wherein heteronuclear or homonuclear metal pairs are effectively coordinated through phthalocyanine ligands, utilizing -F-, -OH-, and -NH_2_- groups as the anchoring sites for metal coordination. FeCo, NiCo, CuCo, and FeCu heteronuclear dual single-atom catalysts (DSACs), and CuCu and CoCo homonuclear DSACs can be obtained via this method.

#### 3.1.2. Spatial Confinement

Spatial confinement is an effective approach for stabilizing SAs by trapping them within nanoscale cavities or embedding them into the lattice of the support materials [108]. Porous materials, such as metal–organic frameworks (MOFs), are used to encapsulate metal precursors, thereby preventing their migration and aggregation. Porous materials enable the in situ construction of a confined microenvironment, providing spatial constraints that regulate the nucleation, growth, and dispersion of metal precursors during synthesis. The isolated metal atoms are stabilized through strong interaction with the support matrix, which suppresses particle agglomeration driven by Ostwald ripening, such as coordination with heteroatoms (N, S, O), resulting in atomically dispersed active sites with a well-defined coordination environment [109]. Moreover, this strategy substantially enhances the metal loading capacity, thereby increasing the density of accessible active sites [110].

SACs integrated with nanoconfinement structures, such as isolated atoms anchored within porous carbon spheres or layered materials, can further enhance pollutant degradation by increasing the local concentration of reactants at the active centers and regulating the diffusion behavior of ROSs. For instance, Shi et al. synthesized Fe single atoms anchored within hollow carbon spheres using silica nanospheres as a sacrificial template [68]. This nanoconfined structure effectively facilitated the simultaneous generation of ^•^OH and ^1^O_2_, significantly boosting catalytic performance. Yang et al. [101] employed mesoporous KIT-6 as a hard template and introduced transition metal salts, salicylic acid, and polyvinylpyrrolidone precursors via impregnation, enabling the self-assembly of a supramolecular structure within the template’s pores. Upon pyrolysis (973 K), carbonization of the precursors led to the formation of hollow carbon nanotubes that confined metal atom mobility and facilitated the formation of atomically dispersed Co-Zn dual sites. Notably, this method requires an acid etching step to remove both the hard template and residual metal clusters. Additionally, adding stabilizers, such as polyvinylpyrrolidone (PVP), can encapsulate metal nanoparticles and improve their dispersibility in metal–organic frameworks (MOFs) [111]. Zhang et al. developed a yolk–shell nanoreactor with dual active sites by pyrolyzing a MOF@SiO_2_ precursor under an NH_3_ atmosphere [112]. Specifically, the precursor was constructed through the self-assembly of Co(NO_3_)_2_·6H_2_O, 2-methylimidazole, and tetraethyl orthosilicate, followed by thermal treatment in NH_3_ to form a yolk–shell nanoconfined structure. In this architecture, the N-doped carbon within the SiO_2_ shell and the CoN species located on the yolk serve as synergistic active sites, significantly enhancing the activation efficiency of peroxymonosulfate (PMS).

#### 3.1.3. CVD

The CVD strategy is applied by introducing gaseous reactants, vaporizing liquids, or solid chemicals to react and deposit on a substrate surface [113]. The coordination environment of single atoms can be precisely tailored by adjusting precursors, temperature, and substrate functional groups. Hydrocarbons, such as alkanes [102], alkenes [114], aromatic hydrocarbons [115], and alcohols [116], as well as polymers, such as polymethyl methacrylate [117] and polystyrene [118], can be used as the carbon sources to yield graphene via CVD in the presence of other organic carbon sources or reducing gases. Additionally, heteroatom-containing organic carbon sources, such as pyridine, ammonia, and borane, can produce doped graphene. Transition metal elements can be introduced by adding highly volatile metal halides and metal–organic compound precursors.

Shen et al. [119] employed ferrocene as the metal precursor and a three-dimensional honeycomb aerogel as the support to synthesize a porous, N-doped carbon (PNC) aerogel electrocatalyst anchored with atomically dispersed Fe-N_4_ sites. Abundant micropores created by Cd volatilization introduced defects that facilitated high-density Fe-N_4_ incorporation, and the Fe-SA@PNC exhibited remarkable ORR activity in an alkaline solution. Hu et al. [120] employed an ingenious CVD strategy with stepwise thermal ramping to fabricate coaxial Co-N-C single-atom catalysts with robust metal-ion anchoring and uniform atomic dispersion, which possess dramatically enhanced 2e^−^ ORR activity. Lu et al. [99] synthesized epoxy-group-functionalized Co-N-C materials via CVD followed by an oxidation process, thereby facilitating H_2_O_2_ generation at the Co sites. During thermal processing under an H_2_ atmosphere, Co(NO_3_)_2_·6 H_2_O was converted to Co nanoparticles dispersed uniformly on NaCl surfaces. These nanoparticles and the NaCl template act as the nucleation sites and substrate, respectively, enabling simultaneous Co-N incorporation and carbon deposition at the same location and thus forming the Co-N-C architecture.

#### 3.1.4. Carbon-Assisted Flash Joule Heating

Joule heating is a novel, ultrafast, and high-temperature processing method, which utilizes the thermal energy released rapidly from high-voltage discharge to achieve synthesis within seconds to minutes. This approach offers a compelling alternative to conventional furnace-heating methods, overcoming the key limitations, such as high energy consumption, low thermal efficiency, and prolonged reaction time [121]. Governed by Joule’s law, this technique enables precise modulation of voltage, current, resistance, and duration, enabling fine-tuned control over the heat input during synthesis. Detailed classification of Joule heating techniques and the associated apparatuses have been well summarized in a previous work [98].

Xing et al. [122] prepared a three-dimensional porous monolithic graphene doped with single-atom cobalt and N using a transient heating–quenching strategy within 2 s. The rapid Joule heating enables simultaneous graphene reduction and doping of metal and nitrogen atoms. The transient quenching process prevents metal atom aggregation during slow cooling, thereby ensuring the stable atomic dispersion of transition metal active sites within the catalyst. Yao et al. [103] utilized a controllable high-temperature shockwave to synthesize and stabilize single atoms at very high temperatures (1500–2000  K), which was achieved by periodic on–off heating (55 ms heating followed by 550 ms off). A brief heating duration helps minimize metal volatilization at high temperatures and mitigates substrate deterioration. For instance, a kinetic energy barrier needs to be overcome for the dispersed Pt atoms to form Pt-C bonds, which can only be achieved at high temperatures. Following a shockwave at 1500 K, the initially weak interactions are converted into robust Pt-C bonds with higher binding energy and enhanced stability, highlighting the critical role of high temperatures in facilitating the atomic dispersion and anchoring. Moreover, substrate defects play a crucial role in anchoring single atoms and determining their loading density. This strategy also has broad versatility in the synthesis of single-atom catalysts on a range of supports, such as reduced graphene and graphitic carbon nitride.

#### 3.1.5. Biomass-Derived Carbon-Supported SACs

**Figure 5:**
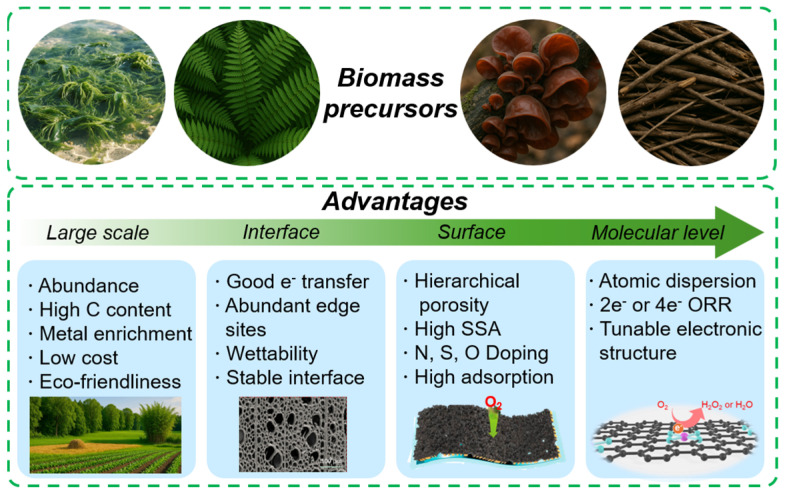
Schematic diagram illustrating the advantages of carbon-supported SACs derived from biomass.

SACs synthesized using biomass precursors, such as microalgae, ferns, *Auricularia judae*, and wood, offer a cost-effective and scalable route for catalyst production, streamlining the fabrication processes and enhancing their applicability in wastewater treatment, as well as in energy conversion and storage systems (Figure 5) [73,123,124,125,126]. The Fe-C and Fe-N bonds naturally present in these macromolecules may play a role analogous to the interactions between metal ions and functional groups commonly employed in conventional chemical synthesis routes. Moreover, the inherently low contents of well-dispersed iron in biomass help increase the spatial separation between adjacent metal atoms, thereby favoring the formation of isolated single-atom sites during high-temperature pyrolysis [76,127].

Li et al. utilized ferns grown in iron mines to fabricate an Fe SAC confined in the porous carbon, with the Fe-N_4_ structure significantly enhancing the catalytic activity and stability [124]. Peng et al. utilized iron- and nitrogen-rich (0.2–0.5 wt% and 8–10 wt%, respectively) *Enteromorpha prolifera* as the precursors to prepare iron-based SACs via pyrolysis [128]. EXAFS measurements revealed that the iron species in the *Enteromorpha*-derived carbon matrix existed predominantly in the form of Fe-N_x_ moieties and nanoscale clusters [128]. The local structure of Fe-N_x_ can be tuned from Fe-N_4_ to Fe-N_6_ with enhanced electron transfer capability, through a co-pyrolysis strategy involving *Enteromorpha prolifera* and polymeric carbon nitride at 1073 K under a high-purity N_2_ atmosphere. The resulting catalyst exhibited exceptional performance in the degradation of atrazine [129].

### 3.2. Modulation Strategies for Catalytic Performance

**Figure 6:**
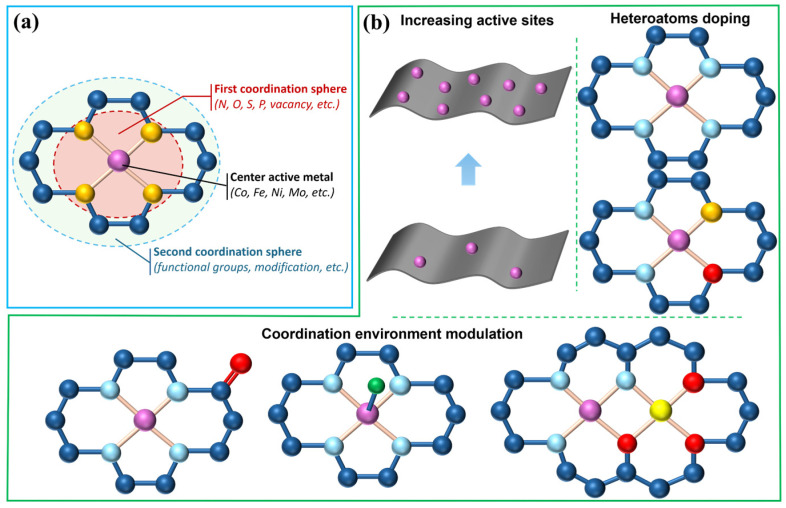
Modulation strategies for catalytic performance: (**a**) schematic of SACs, highlighting the first and second coordination spheres and active metal center; and (**b**) different engineering strategies for modulating the metal catalytic centers (Adapted form [131,133]).

Poor ORR performance or excessive overreduction can limit the degradation efficiency of pollutants [130]. To overcome these limitations, various strategies have been developed to modify pristine carbon materials, aiming to enhance ORR activity and improve the selectivity toward the 2e^−^ reduction pathway. Tang et al. demonstrated that the electrocatalytic behavior is governed not solely by the isolated metal atoms, but also by the synergistic interaction between the metal center and its surrounding first and second coordination spheres [131]. By tuning the coordination environment through ligand engineering around the metal center and functional modification of the host carbon matrix, the catalytic pathway of metal sites can be selectively modulated from an optimal 4e^−^ to a 2e^−^ ORR pathway (Figure 6a). The catalytic performance of SACs can be rationally tailored by precisely tuning the properties of the metal center, the configuration of the adjacent coordination dopant, and the overall metal loading (Figure 6b) [132,133]. Nevertheless, such optimization remains challenging due to the limited availability of versatile synthetic strategies and the incomplete understanding of the structure–property relationship.

#### 3.2.1. Increasing Active Sites

Achieving high metal loading in SACs while preserving atomic dispersion remains a significant challenge, primarily due to the intrinsically high surface energy of metal atoms, which drives their tendency to migrate and aggregate into nanoparticles or clusters [134,135]. For most carbon-based SACs, metal loading typically centers around 1 wt%, with some extreme cases reaching up to 7 wt% [136]. Nevertheless, these values remain significantly lower than the theoretical anchoring capacity of the support for metal atoms.

Jiang et al. employed a post-metal replacement strategy to synthesize Fe-N-C SACs using a porphyrin-based MOF containing bimetallic Zn and Fe as the precursor [137]. While direct pyrolysis led to the formation of Fe nanoparticles when Fe loading exceeded 1.33 wt%, the post-replacement method allowed for a higher Fe loading of up to 2.39 wt% without compromising the atomic dispersion. Wang et al. developed a negative-pressure annealing technique and successfully synthesized 13 types of transition metal SACs on PCN support, achieving high metal loading of up to 27.8–44.3 wt% [138]. The negative-pressure environment effectively suppressed metal migration and aggregation, and promoted the formation of M-N bonds. Similarly, Hai et al. used a two-step annealing strategy to obtain high-loading SACs (10–23 wt%) of 15 transition metals using supports rich in anchoring sites, such as N-doped carbon and polymeric carbon nitride. Due to its nitrogen-rich triazine rings, the latter exhibited the highest density of surface metals. In the first low-temperature annealing step, metal precursors bound selectively to the anchoring sites on the support. The unbound species were then removed by washing, followed by high-temperature annealing to eliminate the residual ligands and stabilize the single-atom sites.

#### 3.2.2. Heteroatom Doping

Heteroatom doping not only improves the dispersion of the metal centers but also modulates their electronic structure. An ideal support must offer sufficient binding strength and abundant anchoring sites to effectively stabilize the dispersed metal atoms [134]. However, conventional porous carbon often possesses an inert surface, limiting its ability to immobilize single metal atoms. To overcome this, heteroatoms, such as N, S, O, and P, are commonly introduced to create additional binding sites [139,140]. These dopants, owing to their distinct electronegativity and atomic radii, can further tune the electronic structure and coordination environment of the central metal atom. For instance, Fe-N_x_-C sites are known to favor the 4e^−^ ORR pathway due to the strong adsorption of intermediates, such as O and OH [141], whereas coordination with oxygen heteroatoms can shift the selectivity toward the 2e^−^ pathway, making it the dominant mechanism for oxygen reduction [77,142]. In contrast, Co-N_4_ sites typically exhibit higher selectivity toward the 2e^−^ pathway. The Co center has weaker binding affinity toward the *OOH intermediate than the Fe center, which favors the desorption of *OOH before O-O bond cleavage and thus enhances H_2_O_2_ production [83,143]. By altering the adjacent coordination dopants and the coordination numbers, the catalytic reaction can be flexibly tuned. Detailed understanding of how heteroatom doping modulates the electronic structure and ORR performance of metal active centers is crucial for designing and synthesizing efficient catalysts.

Nitrogen doping (e.g., pyridinic N and graphitic N) enhances the electron density and facilitates metal coordination. Among these N species, M-N_4_ coordination sites typically serve as the primary active centers for the ORR due to their well-defined geometry and strong binding to O_2_ intermediates. N atoms can coordinate with transition metals, such as Fe or Co, to form Fe/Co-N_x_ sites, where x typically ranges from 2 to 4, thereby enhancing the stability and ORR performance of the isolated metal centers [144]. Pyridinic N also contributes significantly by enhancing oxygen adsorption and electron transfer [145,146]. In contrast, graphitic N mainly promotes electron conductivity and facilitates ORR kinetics, while oxidized N and pyrrolic N play more auxiliary roles [147]. For H_2_O_2_ activation, species such as pyridinic N and M-N_4_ have shown favorable electron redistribution capability, enabling efficient decomposition or further transformation of H_2_O_2_ in advanced oxidation systems. It has been shown that N, S co-doped graphene catalysts exhibited promising bifunctional activity for both the generation and activation of H_2_O_2_, even in the absence of metal sites [148].

#### 3.2.3. Modulating the Coordination Environment of Single Metal Stoms

Axial coordination engineering provides a powerful strategy to fine-tune the electronic structure of single metal sites, steering both the selectivity of the ORR toward the 2e^−^ pathway and the subsequent activation of H_2_O_2_ to generate ^•^OH, thereby enabling efficient cascade AOP processes. Highly electronegative atoms, such as Cl and O, are capable of stabilizing cationic intermediates by delocalizing positive charge. For instance, FeN_3_Cl active sites featuring axial Fe-Cl coordination were found to exhibit superior performance compared to the conventional FeN_4_ configuration [149]. This coordination environment significantly reduces the energy barrier of the ORR at the Fe center, thereby enhancing reaction kinetics. The catalyst had a half-wave potential of 0.81 V in acidic media and 0.91 V in alkaline media. Ren et al. [61] designed Fe-Cl_2_C_2_ and Fe-Cl_2_C_3_ coordination configurations using Cl heteroatom doping. Characterization and DFT calculations reveal that the Fe(III) state (associated with Fe-Cl_2_C_3_) effectively promotes H_2_O_2_ generation via the 2e^−^ pathway, while the Fe(II) state (associated with Fe- Cl_2_C_2_) plays a vital role in activating the transformation of H_2_O_2_ into reactive ^•^OH. Such modulation facilitates a synergistic and efficient 3e^−^ pathway for the conversion of O_2_ to ^•^OH. This catalyst could achieve 98.12% amoxicillin removal within 15 min, largely due to the significantly enhanced yield of ^•^OH.

It is well recognized that DACs can facilitate tandem catalysis and bifunctional site design through the induced synergistic effect [150,151]. Non-precious metal-based catalysts featuring heteronuclear double active sites with two metal centers can interact independently with different reaction intermediates and catalyze sequential steps, thereby significantly enhancing both the activity and selectivity in multi-step catalytic processes [48]. For instance, FeCo-N_6_ sites with atomic distance of 2.58 Å exhibit stronger ORR performance than NiCo-N_6_, owing to the favorable charge transfer occurring between adjacent Fe and Co atoms [152]. Chen et al. reported that the Mo atom can facilitate electron transfer from organic pollutants with high ionization potentials to adjacent Co sites, thereby enhancing the catalytic degradation performance of DACs [45].

Specific oxygen-containing functional groups are expected to coordinate with metal precursors [153]. To precisely control the metal loading in SACs, Yan et al. [154] developed a confined atomic layer deposition strategy to prepare catalysts with stable high loadings of Co single atoms supported on graphene with vacancy (Co_1_/G SACs). Reduced graphene oxide was selected as the substrate, and oxygen-decorated carbon sites were introduced via combustion in an O_3_ atmosphere. By tuning the number of ozone treatments, they modulated the density of pre-coordinated oxygen-containing functional groups on the reduced graphene oxide, enabling controlled synthesis of Co/G SACs with Co loadings ranging from 0.4 to 2.5 wt%. Aberration-corrected STEM-ADF imaging confirmed the atomic dispersion of Co species, with no observable clusters or nanoparticles even at high loadings. EELS and XAFS characterization, together with DFT calculations, revealed that the isolated Co atoms adopt a Co_1_-O_2_C_4_ hexacoordinated configuration, anchored at vacancy sites through strong Co-O interactions. This approach offers a versatile and scalable route to tailor the atomic configuration and metal content in SACs and holds significant promise for the rational design of high-performance electrocatalysts.

## 4. Environmental Factors Influencing Cascade ORR

### 4.1. Inorganic Anions

The type and concentration of anions can change the surface tension of the solution and affect the stability of the catalyst. High ionic strength compresses the electrical double layer at the interface, which can increase or decrease the catalyst’s zeta potential [155]. In general, the addition of Cl^−^ and SO_4_^2−^ increases the conductivity of the solution, which can enhance the removal rate [156]. In contrast, PO_4_^3−^ and NO_3_^−^ may occupy the active sites on the catalyst surface, thereby inhibiting degradation efficiency. The presence of SCN^−^ can lead to the deactivation of metal active sites [42]. Xu et al. reported that the rate of interfacial electron transfer was enhanced in high-SO_4_^2−^ solutions, which significantly improved the degradation efficiency of organic pollutants. However, due to the non-selective oxidation of ^•^OH, common anions in wastewater can adversely affect pollutant degradation [157,158]. For example, Cl^−^ rapidly reacts with ^•^OH to form secondary radicals with weaker oxidative potential (^•^Cl, E_(·Cl/Cl_^−^_)_ = 2.55 V) and thus inhibits pollutant degradation [159,160].

### 4.2. Natural Organic Matter (NOM)

NOM is widely present in surface water and groundwater, typically at concentrations ranging from 1 to 10 ppm, and often reaching 5 to 30 ppm in most types of wastewater [161]. The structure of NOM commonly contains functional moieties, such as phenols, quinones, olefins, and amines, which can chelate with metal centers of SACs, block active sites, and hinder the adsorption of reactive oxygen intermediates [162]. In the presence of NOM, competition with target pollutants for ^•^OH often leads to significant reduction in pollutant degradation efficiency. Jiang et al. reported that the degradation efficiency of florfenicol decreased by 50% in an electro-Fenton system when the concentration of humic acid reached 10 ppm [163]. In contrast, for non-radical systems where ^1^O_2_ is the primary reactive species, the presence of NOM, even at concentrations as high as 50 ppm, did not significantly affect the degradation of target pollutants [71].

### 4.3. pH

**Figure 7:**
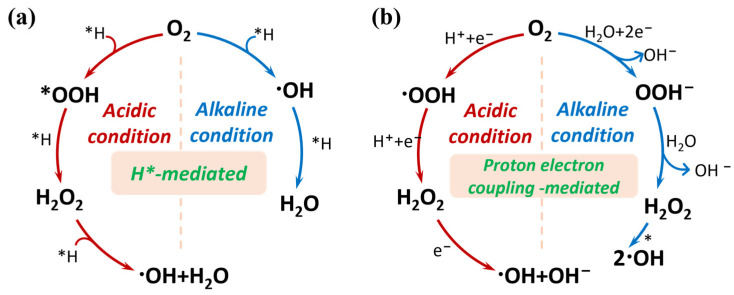
Schematic illustration of 3e^−^ ORR pathways: (**a**) *H-mediated pathway; and (**b**) proton electron coupling-mediated pathway.

The proton-driven 3e^−^ reduction pathway occurs predominantly at noble metal active sites. Notably, the formation of atomic hydrogen in this process is largely independent of solution pH (Figure 7a). Under acidic conditions, atomic hydrogen is generated via proton reduction, whereas it originates from reduction of H_2_O in neutral or alkaline media. Therefore, the decomposition of H_2_O_2_ via this pathway can be sustained over a broad pH range [157,164]. In contrast, the electron-driven 3e^−^ reduction mechanism proceeds through a distinct route, involving direct electron transfer and intermediate species (Figure 7b).

Under mildly alkaline conditions, H_2_O_2_ can undergo self-decomposition to produce ^•^OH. This inherent property makes transition metal catalysts exhibit enhanced activity and stability under mildly alkaline conditions [86]. Song et al. [27] fabricated an Fe SAC that exhibited excellent H_2_O_2_ selectivity, achieving a yield of 86% under strongly alkaline conditions (pH 13). Fe atoms embedded in pyridine-N sites were more effective in both producing and activating H_2_O_2_ than those coordinated with O atom sites. While H_2_O_2_ is more stable in an acidic medium, its concentration is higher under alkaline conditions, suggesting that alkaline media are more favorable for its generation in this system. The radicals can be produced through both the self-decomposition of H_2_O_2_ and its activation driven by the Fe^3+^/Fe^2+^ redox cycle. After five consecutive cycles, Fe leaching was limited to only 1.74 mg, and the chloramphenicol removal efficiency remained as high as 95.5%, demonstrating the excellent durability and practical applicability of the Fe SAC in wastewater treatment applications.

Under circumneutral conditions, the limited regeneration of Fe^2+^ hinders efficient production of ^•^OH. However, in SAC-based systems, this limitation can be overcome by tailoring the coordination environment of active sites [153]. The catalytic performance under neutral conditions can be further enhanced by introducing specific functional groups. For instance, Pang et al. [41] investigated the H_2_O_2_ selectivity of MCHD-9:1, which featured suitable mixed oxygen and COOH functional groups, in 0.1 M KOH (pH 13), 0.1 M phosphate buffer (pH 7), and 0.5 M H_2_SO_4_ (pH 1). The catalyst exhibited the fastest ORR kinetics under alkaline conditions, where a moderate ring current indicated the coexistence of both 4e^−^ and 2e^−^ pathways. Notably, under neutral pH, it exhibited a moderate ORR onset potential and the highest H_2_O_2_ selectivity of 99.9% at 0.57 V vs. RHE, indicating its potential for a cascade ORR under mild conditions. A similar trend was also observed by Pan et al. [15]. Despite these advances, challenges still exist under neutral conditions due to the limited proton availability and poor electrolyte conductivity, which restrict ORR activity. Modulation of the highly tunable electronic structure of SACs shows promise for overcoming such intrinsic limitations associated with neutral media and enables efficient ORR performance.

The generation and subsequent activation of H_2_O_2_ under acidic media remain challenging due to its intrinsic chemical stability at low pH, limiting its effective conversion into ^•^OH. Additionally, Nafion, which is commonly employed as a proton-conducting electrolyte or binder during the cathode coating process, tends to further stabilize H_2_O_2_ rather than facilitate its activation in acidic media [165]. These factors underscore both the significance and challenges in developing high-performance electrocatalysts capable of an efficient cascade ORR under acidic conditions. Recent studies have demonstrated that self-supported electrodes fabricated from conductive substrates, such as carbon felt and nickel foam loaded with transition metal SACs, exhibited remarkable cascade ORR performance in acidic media [166,167,168]. Compared with conventional powder materials, these binder-free electrodes not only possessed reduced internal resistance and improved electron transfer efficiency, but also effectively mitigated catalyst loss, thereby significantly enhancing their stability and practical applicability.

## 5. Stability Challenges and Material Solutions

While extensive efforts have been devoted to enhancing the activity and selectivity of 3*d* transition metal (TM_3d_) SACs in electrocatalysis, the quick degradation in their performance under operating conditions has received increasing attention. The generated ROSs with strong oxidation power could damage the active sites of SCAs, resulting in the loss of catalyst activity or even deactivation. Various degradation pathways have been identified for TM_3d_-N-C SACs, including demetallation (the detachment or dissolution of single metal atoms from the support), carbon corrosion (oxidation and degradation of the carbon support structure), active site poisoning (adsorption of inhibitors or byproducts onto the active sites), micropore flooding (blockage of active sites by other molecules), and fouling (deposition of organic and inorganic matter from the complex wastewater matrix on the catalyst surface) [169]. Among these, demetallation and carbon corrosion have been extensively investigated due to their predominant contribution to the performance decline [170,171]. Various material-based solutions have been proposed to enhance the stability of SACs, as summarized below.

### 5.1. Enhancing the Metal–Support Interaction

Constructing metal–N or metal–O coordination sites can effectively reduce catalyst deactivation [172]. Zhu et al. reported that little metal-ion leaching was detected from FeCo-N DACs within the pH range of 3 to 10, indicating that strong N coordination effectively suppressed demetallation [173]. Under the optimal operating conditions, 20 mg DACs allowed efficient degradation of sulfamethoxazole (78.96 μmol·L^−1^) for up to 8.33 h with zero metal release, and this operational lifetime is much longer than those of Co_3_O_4_ and Fe_3_O_4_.

### 5.2. Spatial Confinement Strategy

Confining metal centers within microporous or mesoporous carbon frameworks can limit their migration and aggregation [174]. Hollow carbon spheres, yolk–shell architectures, and layered structures provide nanoconfined environments that enhance metal dispersion and protect the active sites from external stressors [175]. Such confinement also facilitates the accumulation and local conversion of ROSs, improving both performance and durability [112].

### 5.3. Constructing Self-Supporting Cathodes

Self-supported structures, such as metal-loaded carbon felts or nickel foams, improve mechanical strength and electron transport while eliminating the need for polymeric binders, which can impede mass transfer and promote delamination [176]. These binder-free architectures reduce the internal resistance and exhibit enhanced long-term stability under acidic and neutral conditions, making them promising candidates for practical applications [177].

### 5.4. Combining with More Stable Metal Centers

SACs based on 4*d* and 5*d* transition metals show enhanced stability under harsh conditions due to their inert electronic configurations and weak interaction with H_2_O_2_, reducing Fenton-like degradation of substrate [178,179]. Qin et al. reported that S-coordinated Ru-N-C catalysts outperformed commercial Pt/C in durability [180,181]. Therefore, combining TM_3d_ and TM_4d/5d_ into DACs may enhance the selective 3e^−^ ORR while improving structural integrity, offering a promising solution to balance catalyst efficiency and durability.

## 6. Recommendations for Future Research

The preceding sections summarized the recent advances in cascade ORRs mediated by atomically dispersed transition metal–carbon cathodes for wastewater treatment. Despite notable progress, key challenges remain, including limited understanding of atomic-level electron transfer and the structure–performance relationship of active sites. Practical application is further hindered by the challenges in achieving high metal loading with stable atomic dispersion and durability under real conditions. Future efforts should focus on scalable synthesis to prevent aggregation, in situ techniques to probe mechanisms, and the design of catalysts that balance activity, selectivity, and stability. Addressing these gaps is essential for translating lab-scale advances into effective wastewater treatment technologies. The below directions are recommended for future research.

### 6.1. Date-Driven Catalyst Design

The complexity of SAC structures and reaction pathways makes the design of efficient catalysts a challenging task. Coupling DFT calculations with machine learning enables efficient screening of active sites and prediction of catalytic performance. For instance, Guo et al. identified SACs with optimized HOO*- and O*-binding energy by means of density functional theory, with the fabricated catalysts possessing both high activity and selectivity [82]. Hence, developing reliable descriptors and structure–reactivity models will accelerate the discovery of SACs tailored for specific ORR pathways and pollutant targets.

### 6.2. Balancing Performance and Stability/Durability

Enhancing the activity of SACs for cascade ORRs often compromises their structural integrity under operational conditions. Single-atom sites, especially those based on TM_3d_, are vulnerable to demetallation, agglomeration, and support corrosion in the presence of reactive oxygen species [169]. Strengthening metal–support interactions through optimized M-N/O coordination and spatial confinement has proven effective in mitigating these issues [175,177]. However, a more comprehensive understanding of the degradation mechanism and the dynamic evolution of active sites during electrochemical treatment is still needed to guide the design of SACs that are both highly active and intrinsically stable.

### 6.3. Optimizing Reactors for Efficient Mass Transfer

Reactor configuration significantly influences mass transfer, catalyst utilization, and thus overall system efficiency. Gas diffusion electrodes (GDEs), in both filter-type and contact-type forms, have demonstrated potential for enhancing oxygen transport. While filter-type GDEs offer higher reactivity, they are more prone to fouling due to alkaline-induced precipitation near the catalyst–water interface [182]. Contact-type designs, although less efficient in some cases, offer improved operational stability [183]. Future designs should focus on interface engineering, anti-scaling modification, and optimization of gas–liquid–solid contact to maintain high performance for long-term applications.

### 6.4. Integration with Other Technologies

Integrating cascade ORRs with other advanced processes can enhance system robustness and expand treatment scope. Photoelectrocatalytic systems can improve electron transfer and expand ROS generation routes [80,184]. Membrane-based electrochemical reactors offer advantages in selectivity, separation efficiency, and reusability [185]. Such hybrid systems exploit complementary mechanisms, and offer greater flexibility and scalability than the individual ones. Efficient technology integration would lead to the development of modular, high-performance treatment platforms.

### 6.5. Assessing the Risk of Degradation Intermediates

Instead of being fully mineralized into CO_2_ and H_2_O, organic pollutants are often oxidized into a range of degradation intermediates in EAOPs [186]. Depending on their molecular structures, some of these intermediates are more stable and even more toxic than the parent compounds [187]. Thus, the potential toxicity and risk of degradation intermediates deserve attention. Generally, identification of degradation products through untargeted mass spectrometry, validation of degradation pathways via computational chemistry methods, and assessment of ecological and human health risk using molecular docking, toxicity assays, and predictive modeling collectively offer an effective framework for evaluating the potential risk of treated wastewater. Re-design of the catalytic treatment system may be necessary to eliminate the formation of degradation intermediates with elevated risk.

## Figures and Tables

**Table 1:** Summary of recent studies on use of cascade ORR to treat organic pollutants in heterogeneous electro-Fenton system.

Catalyst	Operation Conditions	Active Sites	Pollutant	Treatment Performance *	Major ROSs	Ref.
FeN_6_/CN	0.025 mol·L^−1^ Na_2_SO_4_, potential: −0.8 V vs. Ag/AgCl, pH 6	FeN_6_	0.1 mmol·L^−1^ 4-CP	90% ΔD and 59% ΔT within 120 min	^1^O_2_, ^•^OH	[28]
Ni_0.5_-Fe_0.5_-NC	0.1 mol·L^−1^ Na_2_SO_4_, potential: −0.5 V vs. SCE, pH 7	FeNi-N_6_	55.9 μmol·L^−1^ florfenicol	67.75% ΔT within 20 min	^•^OH	[51]
FeCo@NPC	Potential: −0.7 V vs. Ag/AgCl, pH 7	FeCo-N_8_	phthalate	93%ΔD and 46% ΔT within 300 min	^•^OH, ^•^O_2_^−^,	[58]
FeCMs	15 mg/L Na_2_SO_4_	Fe-N_4_	5.74 mmol·L^−1^ dimethylacetamide	42.1% ΔD and 3.1% ΔT within 10 min	^•^OH	[59]
FeCuSA-NPC	0.05 mol·L^−1^ Na_2_SO_4_	Fe-N_4_, Cu-N_4_	15.6 μmol·L^−1^ 4-CP	95% ΔD and 41% ΔT within 60 min	^•^OH	[60]
FeCl_2_C_x_/PC	0.05 mol·L^−1^ Na_2_SO_4_, pH 7.41, current density: 15 mA cm^−2^	Fe-Cl_2_C_2_	2.74 μmol·L^−1^ amoxicillin	98.12% ΔD and 62.5% ΔC within 15 min	^•^OH	[61]
SAFe@HSC	0.1 mol·L^−1^ K_2_SO_4_, current density: 20 mA·cm^−2^, pH 7	Fe-N_4_	56.0 μmol ·L^−1^ Thiamphenicol	100% ΔD and 67.8% ΔT within 40 min	^•^OH, ^•^O_2_^−^	[62]
CoFe DAC	0.1 mol·L^−1^ Na_2_SO_4_, potential: −0.6 V vs. SCE, pH 6	Fe-N_4_, Co-N_4_	53.1 μmol·L^−1^ phenol	100% ΔD and 87.2% ΔT within 120 min	^•^OH	[16]
Cu-N@C-700	0.05 mol·L^−1^ Na_2_SO_4_, current density: 50 mA·cm^−2^	Cu^0^/Cu^+^/Cu^2+^ cycle	60.0 μmol·L^−1^ pefloxacin	100% ΔD and 48.6% ΔT within 60 min	^•^OH	[24]
FeN_2_O_2_	0.05 mol·L^−1^ Na_2_SO_4_, potential: −0.4 V vs. SCE, pH 7	Fe-N_2_O_2_	213 μmol·L^−1^ phenol	100% ΔD and 75.2% ΔT within 90 min	^•^OH	[63]
FeCu/NC	0.05 mol·L^−1^ Na_2_SO_4_, current density: 33 mA·cm^−2^, pH 5.9	-	39.7 μmol·L^−1^ lisinopril	100% ΔD and 37.1% ΔT within 120 min	^•^OH	[64]
Boron-modified porous carbon	0.05 mol·L^−1^ Na_2_SO_4_, current density: 33 mA·cm^−2^	C=O and BC_2_O act on H_2_O_2_ generation, BCO_2_ acts on ^•^OH generation	319 μmol·L^−1^ phenol	100% ΔD within 20 min and 73% ΔC within 180 min	^•^OH	[65]
CuBN-HCMs	0.1 mol·L^−1^ Na_2_SO_4_, potential: −0.4 to −0.7 V vs. Ag/AgCl, pH 1–9	CuN_4_-B	213 μmol·L^−1^ phenol	100% ΔD within 60 min and 74.8% ΔC within 180 min	^1^O_2_, ^•^OH	[66]
B-Fe@BC	0.05 mol·L^−1^ Na_2_SO_4_	-	106 μmol·L^−1^ phenol, 39.5 μmol·L^−1^ sulfamethoxazole	100% within 40 min and 70.7% ΔC within 60 min	^1^O_2_, ^•^OH	[67]
Fe_x_HCS	0.1 mol·L^−1^ Na_2_SO_4_, potential: 0 V vs. RHE, pH 7	-	55.4 μmol·L^−1^ ofloxacin	72.7% ΔT within 60 min	^1^O_2_, ^•^OH	[68]
Fe_2_Co_1_/NPC	0.05 mol·L^−1^ Na_2_SO_4_, current density: 100 mA·cm^−2^, pH 7	-	45.0 μmol·L^−1^ tetracycline	91% ΔD within 60 min	^•^OH	[69]
Co_2_-NC/Fe_3_-C_3_N_4_	0.05 mol·L^−1^ Na_2_SO_4_, current density: 10 mA·cm^−2^, pH 7	Fe-N_4_, Co-N/O_5_	97.0 μmol·L^−1^ ibuprofen	93.1% ΔD and 58.8% ΔT within 60 min	^•^OH	[70]
Self-supporting N@C-CC	0.05 mol·L^−1^ Na_2_SO_4_, potential: −0.7 V vs. SCE, pH 7	N-C	0.100 μmol·L^−1^ phenol	100% ΔD within 60 min and 75.5% ΔT within 180 min	^1^O_2_	[29]
Cu-C aerogel	0.05 mol·L^−1^ Na_2_SO_4_, current density: 2.5 mA·cm^−2^, pH 7	CuN_4_	43.8 μmol·L^−1^ bisphenol A	100% ΔD within 30 min and 72.3% ΔT within 120 min	^1^O_2_	[71]
FeP@ECC	0.05 mol·L^−1^ Na_2_SO_4_, current density: 50 mA·cm^−2^, pH 7	Fe^3+^/Fe^2+^	79.0 μmol·L^−1^ sulfamethoxazole	100% ΔD within 20 min	^•^OH	[72]
N, P self-doped carbon	0.05 mol·L^−1^ Na_2_SO_4_, current density: 20 mA·cm^−2^, pH 7	-	90.0 μmol·L^−1^ tetracycline	7.97 mM H_2_O_2_ produced within 60 min	^•^OH	[73]
SA-FeNGA/CF	0.05 mol·L^−1^ Na_2_SO_4_, current density: 1–5 mA·cm^−2^	Fe-N_x_	1.063 mmol·L^−1^ phenol	100% ΔD within 120 min and 95.5% ΔT within 240 min	^•^OH	[74]
Fe-NSC	0.05 mol·L^−1^ Na_2_SO_4_, potential: −0.6 V vs. Ag/AgCl, pH 6	Fe-N_4_	213 μmol·L^−1^ phenol	100% ΔD and 56% ΔT within 50 min	^1^O_2_, ^•^OH	[75]
Endogenous iron-enriched biochar @Ni-Foam	0.1 mol·L^−1^ Na_2_SO_4_, current density: 30 mA·cm^−2^, pH 3	Fe^3+^/Fe^2+^	151 μmol·L^−1^ ciprofloxacin	86% ΔD within 150 min	^•^OH	[76]

Note: *—ΔD, ΔT, and ΔC represent the degradation rate, mineralization rate, and chemical oxygen demand (COD) removal rate of organic pollutants, respectively.
